# *Aspergillus fumigatus *allergen expression is coordinately regulated in response to hydrogen peroxide and cyclic AMP

**DOI:** 10.1186/1476-7961-8-15

**Published:** 2010-11-03

**Authors:** Marcin G Fraczek, Rifat Rashid, Marian Denson, David W Denning, Paul Bowyer

**Affiliations:** 1 School of Translational Medicine, Faculty of Medicine and Human Sciences, Education and Research Centre (2nd floor), The University of Manchester, Manchester Academic Health Science Centre, NIHR Translational Research Facility in Respiratory Medicine, University Hospital of South Manchester NHS Foundation Trust, Manchester, M23 9LT, UK

## Abstract

**Background:**

*A. fumigatus *has been associated with a wide spectrum of allergic disorders such as ABPA or SAFS. It is poorly understood what allergens in particular are being expressed during fungal invasion and which are responsible for stimulation of immune responses. Study of the dynamics of allergen production by fungi may lead to insights into how allergens are presented to the immune system.

**Methods:**

Expression of 17 *A. fumigatus *allergen genes was examined in response to various culture conditions and stimuli as well as in the presence of macrophages in order to mimic conditions encountered in the lung.

**Results:**

Expression of 14/17 allergen genes was strongly induced by oxidative stress caused by hydrogen peroxide (Asp f 1, -2, -4, -5, -6, -7, -8, -10, -13, -17 and -18, all >10-fold and Asp f 11, -12, and -22, 5-10-fold) and 16/17 allergen genes were repressed in the presence of cAMP. The 4 protease allergen genes (Asp f -5, -10, -13 and -18) were expressed at very low levels compared to the comparator (*β*-tubulin) under all other conditions examined. Mild heat shock, anoxia, lipid and presence of macrophages did not result in coordinated changes in allergen gene expression. Growth on lipid as sole carbon source contributed to the moderate induction of most of the allergen genes. Heat shock (37°C > 42°C) caused moderate repression in 11/17 genes (Asp f 1, -2, -4, -5, -6, -9, -10, -13, -17, -18 and -23) (2- to 9-fold), which was mostly evident for Asp f 1 and -9 (~9-fold). Anaerobic stress led to moderate induction of 13/17 genes (1.1 to 4-fold) with one, Asp f 8 induced over 10-fold when grown under mineral oil. Complex changes were seen in gene expression during co-culture of *A. fumigatus *with macrophages.

**Conclusions:**

Remarkable coordination of allergen gene expression in response to a specific condition (oxidative stress or the presence of cAMP) has been observed, implying that a single biological stimulus may play a role in allergen gene regulation. Interdiction of a putative allergen expression induction signalling pathway might provide a novel therapy for treatment of fungal allergy.

## Introduction

Allergy is becoming one of the most common ailments in the developed world [1,2]. This condition arises from disproportionate IgE-mediated and/or eosinophilic responses of the immune system to contact with an antigen [3]. Such antigens are usually proteins and are termed allergens. The characteristics of allergens that make them allergenic are not well understood but it is considered likely that such proteins must be stable and resistant to proteases or that they possess cryptic structural features that are particularly provocative to the immune system [4,5]. A significant proportion of allergy is caused by fungal proteins [6]. In contrast to other more common environmental allergens such as those from dust mite faeces, pollen or pet dander, fungal allergens are likely to be dynamically expressed by the fungus during transient or long-term colonization of the airways and other mucosal surfaces. Study of the timing and context of gene expression may therefore lead to insights into important events in the early interaction between the immune system and the allergenic protein. In particular, the timing and level of allergen expression may possibly be involved in determining that the protein is an allergen by determining when the protein comes into contact with the immune system for example after macrophage phagocytosis or during interaction with neutrophils or eosinophils. The most common route of exposure to fungi is via the respiratory tract [7-10] although some fungi that produce allergens are also dermatophytes [11]. In the scenario where allergen gene expression is critical to allergenicity of the expressed protein, allergen genes may be expressed constitutively at high levels. Alternatively several possible conditions may be encountered in the lung that may trigger high levels of allergen gene expression. These conditions might be expected to include presence of lung surfactant lipid as a carbon source, anaerobic growth in regions of the lung blocked off by mucus plugs, oxidative stress during phagocytosis, heat shock from inflammatory responses or the presence of immune cells such as macrophages [12-16]. *Aspergillus fumigatus *is well studied as an invasive pathogen of humans [17-19] but is also a major source of fungal allergens involved in allergy and exacerbations of asthma such as Severe Asthma with Fungal Sensitisation (SAFS) and Allergic Bronchopulmonary Aspergillosis (ABPA) [20-23]. The demonstration of coordinated regulation of allergen expression would suggest a possible new therapeutic avenue based in interdiction of a common transcriptional activation mechanism during colonisation. However, few detailed studies on allergen expression have yet been performed. We recently refined the gene structure and classification of the *A. fumigatus *allergens [24,25]. Here we have developed Real-Time PCR expression assays for 17 *A. fumigatus *allergen genes (Asp f 1-12, -13, -17, -18, -22 and -23) and tested various defined conditions that might trigger expression (recent *A. fumigatus *allergen gene nomenclature and their identities are presented in [25]).

## Methods

### A. fumigatus strain, media and growth conditions

In order to test what conditions trigger the expression of *A. fumigatus *allergen genes, the fungus was grown in various culture media chosen to mimic the conditions encountered in the lung. Af293 [26] cultures were grown in 200 ml Sabouraud dextrose broth (SB) for 24 h at 37°C with agitation (200 rpm), washed 3 times in *Aspergillus *minimal medium [27] (AMM) and used to inoculate parallel duplicate 50 ml cultures of AMM containing either (i) 1% glucose, (ii) 0.5% phoshotidyl choline, (iii) 1% glucose + 1.8 mM hydrogen peroxide, (iv) 1% glucose + 5 mM menadione and (v) 1% glucose + 5 mM diamide. Concentrations of peroxide, menadione and diamide (Sigma-Aldrich) were chosen to allow >95% normal growth. Other conditions included (vi) a static AMM + 1% glucose culture, degassed under vacuum for 5 minutes then overlaid with 50 ml mineral oil to create anoxic conditions (changing oxygen tension from 140 mm Hg to 14 mm Hg), (vii) AMM + 1% glucose culture grown at 42°C, (viii) AMM + 1 mM dibutyryl cyclic adenosine monophosphate (dbtcAMP) (Sigma-Aldrich), (ix) AMM only and (ix) SB culture. Subsequently, all cultures were grown with agitation (200 rpm) (except condition vi) for 24 h at 37°C (except condition vii) and approximately 2 ml of each sample was collected after 3 h, 6 h, 9 h and 24 h for RNA extraction.

### Generation of macrophages from peripheral blood mononucleocytes and co-culture with A. fumigatus

Since macrophages are one of the first immune cells that come in contact with a pathogen in the respiratory tract [28], allergen gene expression was also tested after challenge of *A. fumigatus *with blood monocyte derived macrophages. Blood was obtained from healthy volunteers (Wythenshawe Hospital, Manchester, UK) and layered over 10 ml Ficoll using a Pasteur pipette in 50 ml sterile tubes. The tubes were centrifuged for 20 min at 800 × *g *at room temperature in a swinging bucket rotor centrifuge and the buffy coat layer containing monocytes and lymphocytes was removed, and transferred to a new sterile tube. The cells were washed once with Dulbecco's Phosphate Buffered Saline (PBS) (Sigma-Aldrich) and centrifuged for 7 min at 800 × *g *at room temperature. The pellet was subsequently washed twice in Dulbecco's PBS and centrifuged for 7 min at 400 × *g *at room temperature. The cells were resuspended in 5 ml of RPMI 1640-L-glutamine containing 10% Foetal Bovine Serum, 100 U/ml penicillin and 0.1 mg/ml streptomycin, and counted under a haemocytometer (diluted 1:1 with trypan blue (Sigma-Aldrich) for viable count). Macrophages were induced by growth for 10-12 days with addition of 4 ng/ml recombinant human granulocyte-macrophage colony stimulating factor (hGM-CSF). Following the incubation, macrophages were counted and Af293 spores were added to the cultures at two different concentrations denoted here as multiplicities of infection (MOI) - 1:200 and 1:2000 spores/macrophage. The fungus was allowed to grow for 24 h or 48 h before hyphae/macrophage samples were collected for RNA extraction. Cultures that had not been inoculated with fungus (macrophage only and RPMI-FBS-PS only) were used as controls.

### Preparation of RNA

Three aliquots consisting of 2 ml of each culture grown in various media and 200 μl of *A. fumigatus*/macrophage cultures were harvested at various time points and used to prepare RNA. RNA was extracted using the FastRNA Pro Red Kit (MPBio) according to the manufacturer's instruction followed by treatment with RQ1 RNase-Free DNase (Promega) and ethanol precipitation. RNA was subsequently quantified by spectrometry.

### Quantitative PCR (qRT-PCR)

All reactions were performed in a Stratagene Mx3005p qRT-PCR machine. Primer concentrations were independently optimized to favour product formation then amplification efficiency for each optimised primer pair was calculated using a 2-fold dilution series. Twenty five microliter reactions containing 12.5 μl Brilliant II SYBR Green QRT-PCR Mix (Stratagene), RT (Reverse Transcriptase)/RNase block enzyme mixture, intron spanning primers (Table 1) and 100 ng total RNA were cycled at 50°C for 1 h followed by 40 cycles of 94°C for 30 sec, 55°C for 30 sec and 72°C for 60 sec. Fluorescence was read at 55°C three times during each cycle. Melting curves were subsequently determined for each reaction to ensure that single products were produced and the resulting reaction was run on a 1.8% agarose gel to confirm the product was unique and of the correct size. Triplicate or quadruplicate RNA preps from three replicate growth conditions were subjected to qRT-PCR. No RNA and no RT controls were also included. RNA quantitation was then performed according to the 2ΔΔCt method [29]. Allergen qRT-PCR results were normalised against the *β*-tubulin gene and compared to the expression of the same allergen gene in control conditions. Results are presented as mean values in a histofram with standard errors calculated using GraphPad PRISM 4.0 and significant differences were assessed pairwise using students T-tests with P values <0.05 representing significance.

**Table 1:** Intron spanning primers used in the expression analysis.

Gene	CADRE	Forward	pMol	Reverse	pMol
Asp f 1	AFUA_5G02330	ACGCTCGTGCG*ACCTGGACATGC	40	GCCGTCGGAAAGAGGTGCGTG	20

Asp f 2	AFUA_4G09580	CTGTGCTTTGGAAG*GCTGGGGCGGCCAC	40	GTCTCCATGTGCTCCCAGGGC	40

Asp f 3	AFUA_6G02280	GGGACGACATT*CTCTTCCTCTCCGAC	40	CGCTCGAGAACTCGAGGTGGTTC	40

Asp f 4	AFUA_2G03830	CAGCTCTTCCCACTCCGACAG	40	CTGGGTTCGGTCCTGCCAC	40

Asp f 5	AFUA_8G07080	TACTCACGGTC*TTTCCAACCGAC	40	GCTTCAGACGGATGGCCGTC	40

Asp f 6	AFUA_1G14550	ACTACCTTCAG*TACTTGAACGAC	100	GTACACGTTCATGAATGGGTG	40

Asp f 7	AFUA_4G06670	GCTCCTATCTTCAAGTCCCT	40	CCACACTACGTCCACTTCAC	40

Asp f 8	AFUA_2G10100	ACCTCCAGGAGCTCATCGCCGAG	20	CTCCTCCTTCTTCTCCTCAG	40

Asp f 9	AFUA_1G16190	GAGGTTGACTGG*GAAGTATTG	40	GAAAGTCTCCTGAGGAGTG	10

Asp f 10	AFUA_5G13300	GCGGCATTGCTG*ACACCGGC	40	GCAGGGGAAGACATAACCACCG	40

Asp f 11	AFUA_2G03720	GGTCCTAACAC*CAACGGC	40	GAGCTTCGATCTCCTTGAC	40

Asp f 12	AFUA_5G04170	TGACCAAGGCT*GATTTGATC	40	CAACAAGGTAAGCAGAGTAG	40

Asp f 13	AFUA_4G11800	GAGCGCAGAC*GTTGCCCATG	40	CCTTGTGGGAAATGCTGCCCAG	40

Asp f 17	AFUA_4G03240	ACCATCAACTCCGGTGTCGAC	10	CTTGGAGATGAGGTCGTCG	40

Asp f 18	AFUA_5G09210	CTCCCAAC*CTCCTTGCCTG	40	CTCGGCCTTGTGAACTAG	40

Asp f 22	AFUA_6G06770	CATGATCGTCCCTGA*CTCCGC	40	CACCCTCGTCACCAACGTTG	40

Asp f 23	AFUA_2G11850	GCAGATTACTCC*CATGGGTG	40	GTACAGGGTCTTGCGCAG	40

Actin	AFUA_6G04740	TCATCATGCGCGACAGC	10	CAATCATGATA*CCATGGTGAC	40

*β*-tubulin	AFUA_1G10910	CGACAACGAG*GCTCTGTACG	40	CAACTTGCGCAGATCAGAGTTGAG	40

GpdA	AFUA_5G01970	GGCGAGCTCAAGAACATCCTCGGCTA	20	CTTGGCGATGTAGGCGATAAGGTCGA	20

FksA	AFUA_6G12400	GCTGCGCCCAAG*TCGCCAAATC	40	GAACAACAAGTGGGGCAATG	20

As part of the initial work up for these experiments primer concentrations were optimized so that all allergens could be analysed using a single annealing temperature in the PCR. Primer levels used are indicated. Primers used to establish the most useful comparator are also included.

pMol = pMol primer used per 25 μl reaction mix; * - intron site

## Results

### Selection of a standard comparator gene and validation of qRT-PCR

**Figure 1:**
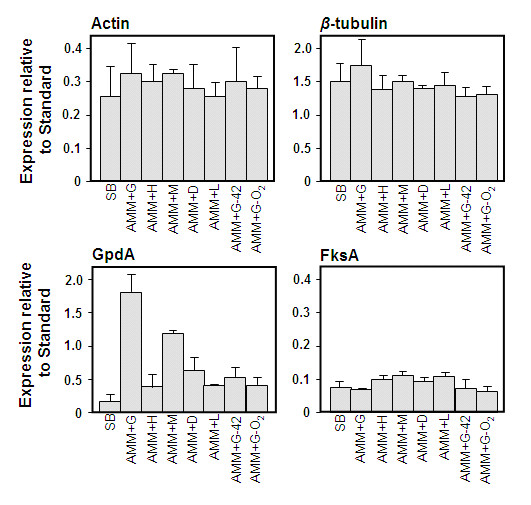
**Relative expression of candidate comparator genes in relation to known standards**. Each gene shown was tested by qRT-PCR on 100 ng RNA from the conditions shown. A 10 pM DNA comparator from the cognate gene was used to estimate relative expression level. Standard errors from triplicate experiments are shown. **SB**, Sabouraud Broth; **AMM**, *Aspergillus *minimal medium; **+G**, +1% glucose; **+H**, +1.8 mM hydrogen peroxide; **+M**, +5 mM menadione; **+D**, +5 mM diamide; **+L**, +1% phoshotidyl choline; **42**, culture grown at 42°C; **-O_2_**, culture grown in anoxic conditions. Error bars represent standard error of mean of biological replicates calculated using GraphPad PRISM 4.0.

Actin, *β*-tubulin, glucan synthetase subunit 1 (Fks1) and glyceraldehyde 3 phosphate dehydrogenase (GpdA) were assessed as controls to normalize expression (accession numbers and the primers are presented in Table 1). cDNA fragments of each gene were isolated by PCR using the primers described then quantified by comparison with known standards on agarose gels and by spectrophotometry. Ten pMol of each cDNA product was used as a comparator against 100 ng total cell RNA to estimate relative RNA levels for each gene under the growth conditions described although direct quantitative comparison between product level from an qRT-PCR reaction and a simple PCR reaction was deemed inappropriate. Actin, *β*-tubulin and Fks1 provided good constitutive controls whereas GpdA was observed to vary considerably in its expression level (Figure 1). As *β*-tubulin appeared to show a useful constitutive level of expression it was used as a standard in subsequent experiments although the actin and Fks1 genes were occasionally used as a "quis custodiet" control to confirm levels of the control comparator. Dissociate curves of qRT-PCR amplified products calculated by plotting the negative derivative of fluorescence [-R´(T)] emitted by the PCR sample during the melting procedure (from 52°C to 95°C) showed a single melting peak with melting temperature (Tm) of 75°C or higher indicating specific qRT-PCR product. Moreover, agarose gel electrophoresis of these products confirmed amplification of a single product for each allergen gene from mRNA and no primer-dimer formation were generated during the qRT-PCR reactions. Control reactions (no RNA and no RT) did not generate any products and no dissociation curves for them were observed (data not shown).

### Relative expression of allergen genes

**Figure 2:**
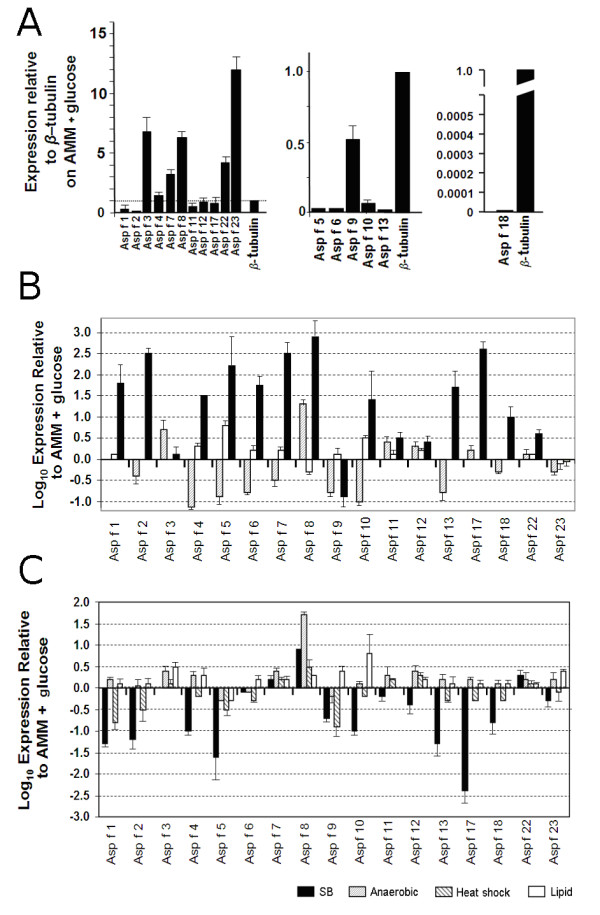
**Expression levels of allergen genes in the presence of different culture conditions**. **A**, expression levels of all allergens relative to a *β*-tubulin comparator during growth on AMM + 1% glucose showing detail of the low expression levels of Asp f 5, -6, -9, -10, -13 and -18 (same data as left panel). **B**, Expression levels of allergen genes in the presence of various oxidative stress inducing agents. Levels are shown on a log scale as expression relative to that observed without addition of oxidative stress inducing agents (AMM + 1% glucose, as shown in panel **A**). **C**, Expression levels of allergen genes under different growth conditions. Levels are shown on a log scale as expression relative to that observed without addition of oxidative stress inducing agents (AMM + 1% glucose, as shown in panel **A**). Error bars represent standard error of mean of biological replicates calculated using GraphPad PRISM 4.0.

In order to test whether allergens are all expressed at high level, relative allergen expression level during growth on AMM containing 1% glucose was determined (Figure 2A). Some allergens, Asp f 3, -7, -8, -22 and -23, showed relatively high level of expression whilst others, notably the proteases Asp f 5, -10, -13 and -18, showed low levels of expression. This basal level expression was used in subsequent experiments to determine whether certain stimuli induced or repressed expression relative to this defined condition.

### Allergen gene expression in response to oxidative stress

The expression of 17 *A. fumigatus *allergen genes was analysed upon fungal growth in various *in vitro *experimental media, chosen to mimic conditions in the lung. As exposure to oxidative stress is reportedly one of the earliest events in fungus host interaction we tested allergen expression in response to hydrogen peroxide, which we expect would be directly encountered by the fungus and menadione and diamide, which alter the internal redox state of the cell [30].

Eleven allergen genes (Asp f 1, -2, -4, -5, -6, -7, -8, -10, -13, -17 and -18) were induced >10 fold compared with control (AMM + 1% glucose) during growth on hydrogen peroxide but not other sources of oxidative stress such as menadione or diamide (Figure 2B). Those allergens include enzymes (among others all 4 tested *A. fumigatus *proteases) and 4 proteins of unknown to date functions (Asp f 2, -4, -7 and -17). Asp f 11, -12 and -22 were induced 5-10 fold under the same conditions. Expression of Asp f 3 (peroxiredoxin) and Asp f 23 (ribosomal L3 protein) was relatively unchanged during growth on hydrogen peroxide. Only one allergen gene, Asp f 9 was repressed under this condition. Thus, the expression of 14/17 allergen genes was induced under a single condition suggesting the possibility of coordinated regulation of allergen gene expression. This type of oxidative stress is similar to that encountered by germinating fungal spores that are engulfed by macrophages with the timing of expression being consistent with reports of the lifespan of engulfed fungal spores [31,32].

Agents that alter intracellular redox balance might be expected to reproduce the effects of exogenous hydrogen peroxide as this is likely to be processed via dismutases and catalases to release intermediates that cause oxidative stress. Alternatively, hydrogen peroxide may play a role in signalling or other cellular processes that is more relevant to the observed allergen induction than simple oxidative damage. Menadione generates superoxide anions (O_2_^·-^) which interact with iron-sulphur clusters in proteins generating hydroxyl radical (OH^·^). Diamide, a thiol-oxidizing agent, results in GSH/GSSG redox imbalance in the cell [30]. Neither compound stimulated the significant induction of allergen genes. On the contrary, 10 genes (Asp f 2, -4, -5, -6, -7, -9, -10, -13, -18 and -23) were repressed during growth on menadione with only one (Asp f 8 coding for ribosomal P2 protein) induced over 10-fold. Diamide marginally increased the expression of most allergen genes (except Asp f 8 and -23), ranging from ~1.1 to ~3 fold. Only Asp f 4, -5 and -10 were induced more than 3-fold, compared to AMM + 1% glucose (Figure 2B).

### Allergen gene expression in response to complex media, lipid, heat shock and anaerobic conditions

Conditions such as anoxia, mild heat shock (42°C) and lipid (phoshotidyl choline) are expected to be encountered by fungi during entry into or colonization of the lung. Here we tested allergen expression levels in response to transfer to media containing these components or growth under these conditions. Samples were analysed at 3 h, 6 h, 9 h and 24 h but only the 9 h time point is presented in Figure 2C for clarity and because little variation in expression was observed between the time points analysed. None of the conditions tested resulted in coordinated high levels of expression or repression of the allergen genes.

Transfer of mycelium from AMM + 1% glucose to complex medium (Sabouraud Broth) caused repression of 12/17 genes tested, except Asp f 3, -6, -7, -8 and -22, however the induction of these genes was not substantial (~1.1 to 3-fold). Seven out of these genes (Asp f 1, -2, -4, -5, -10, -13 and -17) were strongly repressed (over 10-fold) under this condition.

Asp f 5, -6 and -9 were the only genes repressed (4, ~1.1 and ~2 fold, respectively) when grown under mineral oil (anaerobic stress). Asp f 8 was induced over 10-fold and the induction of other genes ranged from 1.5- (Asp f 2) to 5-fold (Asp f 12) under the same condition. Heat shock (42°C) caused moderate repression in 11/17 genes tested (Asp f 1, -2, -4, -5, -6, -9, -10, -13, -17, -18 and -23) with the most evident for Asp f 1 and 9 (~9-fold). Other 6 genes (Asp f 3, -7, -8, -11, -12 and -22) we induced from between 1.5- (Asp f 3) to 5-fold (Asp f 8). Growth on lipid as sole carbon source contributed to the moderate induction of most of the allergen genes. Only Asp f 5 was slightly repressed under this condition (~3 fold).

### Allergen gene expression in response to dibutyryl cyclic AMP

**Figure 3:**
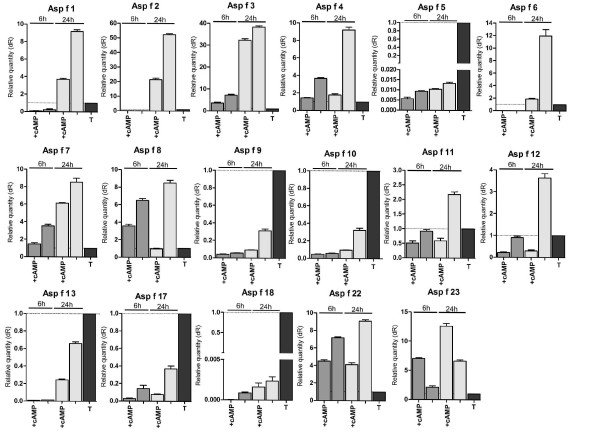
**Effect of cAMP on allergen gene expression**. Relative expression levels on AMM + 1% glucose are shown; 1 mM of dbtcAMP was added to the growth medium and the expression profiles of 17 A. fumigatus allergen genes were assessed by qRT-PCR. The results were normalised against the *β*-tubulin gene and compared to the expression of the same allergen genes in the medium lacking dbtcAMP. Expression of 16/17 allergen genes (except Asp f 23) was repressed by the presence of dbtcAMP; **+cAMP**, expression level on AMM + 1% glucose + 1 mM dbtcAMP; **T**, expression of the *β*-tubulin comparator. Error bars represent standard error of mean of biological replicates calculated using GraphPad PRISM 4.0.

Cyclic AMP is a good candidate signal for control of disparate sets of genes as it acts widely on gene expression in the cell. Allergen qRT-PCR results obtained from the cultures grown in presence of the membrane permeable cAMP analogue dibutyryl cAMP (dbtcAMP) [33] were normalised against the housekeeping gene (*β*-tubulin) and compared to the expression of the same normalised gene in the -dbtcAMP medium after both 6 h and 24 h. Sixteen out of 17 allergen genes tested were significantly repressed in the presence of the dbtcAMP (Figure 3). Only Asp f 23 was induced in the presence of this compound after both 6 h and 24 h. Its expression was higher for both conditions than for *β*-tubulin.

### Response of allergen expression to macrophages

In order to determine whether the coordinated responses observed in axenic culture could be replicated by co-cultivation of *A. fumigatus *with macrophages, expression levels were determined at two different MOI - 1:200 and 1:2000 spores/macrophage. The MOI used were chosen to give conditions where both fungus and macrophage were able to grow and remain viable for the duration of the experiment. Lower MOI (< 1:2000) resulted in complete suppression of the fungus by the macrophages and higher MOI (> 1:200) resulted in rapid overgrowth of the culture with fungus and death of the macrophages after 48 h. To ensure viability, cultures and co-cultures were stained with vital dyes at points throughout the experiment. Microscopic analysis revealed that the macrophages were active and appeared to be aggressively attacking fungal spores throughout. Insufficient RNA was obtained from earlier time points (0 h, 6 h and 12 h) to achieve reproducible results (data not shown) and therefore samples were only analysed after 24 h and 48 h of incubation. The growth form of the fungus was predominantly hyphal at 24 h (> 95% with fewer than 5% spores and germlings remaining). The *Aspergillus*/macrophage qRT-PCR results were normalised against the *β*-tubulin gene and compared to the normalised results obtained from control conditions (*Aspergillus *only) after 24 h and 48 h.

**Figure 4:**
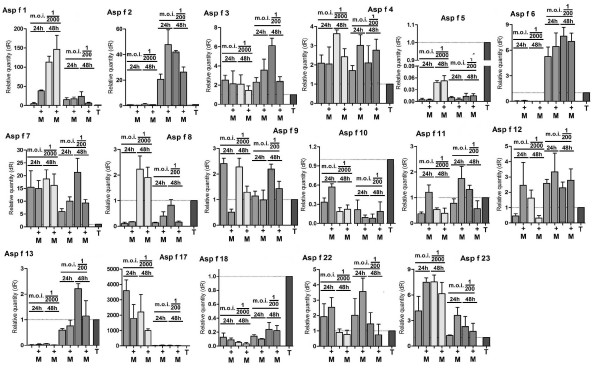
**Expression levels of allergen genes during co-culture with macrophages**. *Aspergillus *was co-cultured with macrophages at two different MOI - 1:200 and 1:2000 spores/macrophage for 48 h. RNA was extracted after 24 h and 48 h and qRT-PCR was used to determine allergen expression levels. Expression levels were calculated for *Aspergillus *exposed to macrophages (**+M) **and *Aspergillus *only samples for both time points. Expression levels are given as units relative to the comparator, *β*-tubulin (**T**) and this is shown for each gene tested to give an indication of level of expression. Error bars represent standard error of mean of biological replicates calculated using GraphPad PRISM 4.0.

The response of allergen expression upon incubation with macrophages is complex and clearly lacks the coordinated nature of the responses observed in axenic culture (Figure 4). The 4 protease allergens, Asp f 5, -10 and -13 and -18 were expressed at very low levels and did not appear to increase expression in the presence of macrophages. The allergen gene expression was strongly affected by the MOI; Asp f 2, -6 and -13 are all strongly expressed at the higher MOI of 1 spore per 200 macrophages but expressed at very low levels at an MOI of 1:2000. In general effects of co-culture age or MOI affected expression whereas presence or absence of macrophages did not alter allergen expression with the exception of Asp f 12. Asp f 9 was repressed by macrophages at an MOI of 1:2000 at both 24 h and 48 h but was not repressed at an MOI of 1:200 although a repressive trend can be observed. Asp f 1 and -11 were induced at 24 h with an MOI of 1:2000 but not affected by presence of macrophages at 48 h or with a higher MOI. Several trends in expression could be imagined from detailed visual inspection of the data.

## Discussion

An increase in incidence of allergy caused by various biological and environmental stimuli has been observed in recent years and *A. fumigatus *has been associated with a wide spectrum of allergic disorders such as ABPA, allergic asthma and SAFS [21,34]. In immunocompetent patients fungal spores are effectively eliminated by macrophages whereas neutrophils are responsible for defence against hyphal fragments [35]. It is hypothesised that regulation of allergen gene expression depends on the environment in which the fungus grows. In this study, *A. fumigatus *allergen expression was tested during fungal exposure to various *in vitro *stimuli, similar to those encountered in the lung as well as during fungal challenge with human immune cells. The experiments presented here show a remarkable coordination of allergen expression in response to growth in the presence of hydrogen peroxide, implying that a single biological stimulus may play a role in gene regulation. Oxidative stress caused by hydrogen peroxide has been shown to strongly induce and dbtcAMP to strongly repress expression of most allergen genes (16/17 for both cases). The up-regulation of allergen genes caused by hydrogen peroxide is consistent with the hypothesis that allergen expression is induced during the release of oxidative agents by macrophages and neutrophils during killing of conidia [35]. This should be especially true for genes coding for proteins, which are involved in conversion of toxic oxidative agents to less toxic compounds, such as Asp f 3 (peroxiredoxin). However, the qRT-PCR data showed that the expression of this gene is only slightly increased by hydrogen peroxide. This may suggest that other mechanisms involved in eliminating peroxide may play a role such as for example activation of genes coding for catalases or glutathione peroxidases, which convert hydrogen peroxide to water [36]. Similar results of limited expression of Asp f 3 were also observed during *A. fumigatus *challenge with human immune cells, however it was dependent on time of incubation and spore concentration used. Since macrophages are able to eliminate spores but not hyphae and because this protein has been localised in germinating spores [37], it is possible that activation of the gene responsible for production of this allergen in hyphae is not as important as it is in spores. However, the expression of allergen genes in conidia was not tested because insufficient RNA concentrations could be obtained. The expression of allergens in response to an oxidative stress stimulus may be highly significant in presentation of the proteins to the immune system via antigen presenting cells such as macrophages or dendritic cells.

It is also evident that exposure to dbtcAMP, which has been shown to be involved in many regulatory processes in various organisms [38], had an effect on expression of *A. fumigatus *allergen genes. Sixteen out of 17 allergen genes were repressed upon addition of this compound (except Asp f 23). Thus, coordinated regulation of allergen gene expression by both cAMP and hydrogen peroxide may suggest that there is a single regulatory pathway, which might be particularly useful in development of possible therapeutic agents in order to control allergic responses.

Several other patterns of allergen gene expression have been observed during *in vitro *experiments. Fungal proteolytic allergens (Asp f 5, -10, -13 and -18) were highly induced during growth in AMM + 1% glucose supplemented with hydrogen peroxide, however results obtained from fungal challenge with human immune cells showed that all of the protease coding genes were highly repressed in such conditions. This confirms the shift in allergen gene expression depending on environmental conditions and suggests that proteases may not be required for the fungus to survive in the presence if macrophages. They might however be highly expressed during growth with bronchial epithelial cells, which contain high levels of protein structural components such as tight junctions [39] or by exposure to mucus containing high level of mucin proteins [40].

The other conditions that most strongly affected gene expression were menadione and anoxia (9/17 and 14/17 allergen genes repressed for these conditions, respectively). Lipid moderately induced expression of 16/17 allergens and complete medium (SB) repressed 12/17 allergen genes tested. It is also evident that the allergen gene expression depends on time of incubation and fungal spore concentration present in the environment. This was confirmed during fungal challenge with macrophages, in which the expression of 11/18 genes (Asp f 1-4, -7, -8, -11, -12, -17, -22 and -23) at the higher MOI (> 1:200) was induced after 24 h but decreased after 48 h of incubation. Since Asp f 1 is a ribotoxin and Asp f 3 is a peroxiredoxin, the up-regulation of genes coding for these proteins was expected. Genes involved in protein synthesis and folding, such as Asp f 8, 11 and 23 were also induced during the first 24 h as expected. In contrast, lower MOI (> 1:2000) caused repression of several allergen genes (Asp f 1, -8, -11 and -23) during the first 24 h of incubation but their induction after 48 h. This suggests that the fungus might activate a putative defence mechanism involving allergen proteins at an early stage of invasion, reducing its gene expression after the host defence mechanism is breached. The progress of conidial germination means that fewer spores and more hyphae will be present in the medium at later time points and because macrophages are only able to eliminate spores [35], the nature of the stress perceived by the fungus might be changed. Sugui and colleagues [41] examined the expression of several conidial and hyphal genes of *A. fumigatus *during exposure to neutrophils and found that the expression of most genes was up-regulated in conidia but not in hyphae. It is therefore possible that after challenge with macrophages, the expression of some allergen genes is induced in conidia and young germlings but not in mature hyphae or vice versa. The AfYAP1 gene of *A. fumigatus *has been shown to be involved in oxidative stress responses and the proteomic analysis presented in this paper lists 28 proteins that are regulated including Asp f 3[42]. No coordinate regulation of allergen genes was observed in an transcriptome study of *A. fumigatus *in an immunocompromised mouse model[43].

The functions of the allergen proteins appear disparate and include proteases, oxidative response proteins, ribosomal components and proteins of unknown role. Therefore, existence of a coordinated expression profile in response to any condition is rather unexpected. Since allergen proteins must interact with the immune machinery at some point in fungal colonization, commensality, clearance or invasion their induction by hydrogen peroxide and repression by cAMP might be reasonably expected to be reflected in the interaction with immune cells. That this assumption is overwhelmingly incorrect in the case of macrophages may be the result of several factors. Firstly, the coordinated responses observed in axenic culture may not be significant in the interaction of fungus and the immune system. However, this seems unlikely given the known involvement of hydrogen peroxide in the early immune response and its demonstrable role in fungal clearance exemplified in chronic granulomatous disease. Secondly, it is possible that macrophages are not important in this respect and that other players in the immune response such as neutrophils, eosinophils or dendritic cells are more significant as sites of allergen induction. Finally we suspect that our co-cultivation experiment suffers from lack of precise spatio-temporal localization of allergen expression and that induction or repression is lost in the averaging effect of large cell numbers, all at different stages in the interaction. We suggest that this would more profitably be studied on the basis of single cell-cell interactions and we are currently examining this avenue by use of GFP-allergen promoter and GFP-allergen protein fusion approaches. Interestingly examination of microarray data from the closely related fungus *A. nidulans *[30] suggests that orthologues of the allergen genes in this fungus are not strongly induced in response to hydrogen peroxide providing a possible explanation for the absence of allergens in this organism.

The coordinated regulation and induction of allergen expression is highly significant as it implies a possible previously unsuspected characteristic of fungal allergen proteins. The disparate allergen proteins may in fact be part of a coordinated response to oxidative attack and that there may be a possible therapeutic route towards reducing or eliminating allergen expression during fungal colonization via interference with sensing and signal transduction of the oxidative stress response. Nevertheless, we note that many proteins are induced in responses to oxidative stress and that relatively few of these become allergens, therefore a role for structural features or physical properties in advancement of a protein to allergenicity seems likely to remain an important consideration.

## Competing interests

The authors declare that they have no competing interests.

## Authors' contributions

PB, MF, RR performed RT-PCR experiments, MD performed macrophage culture. PB, DWD and MF wrote the manuscript. All authors have read and approved the manuscript.
